# Development of a Novel Biosensor Using Cationic Antimicrobial Peptide and Nickel Phthalocyanine Ultrathin Films for Electrochemical Detection of Dopamine

**DOI:** 10.1155/2012/850969

**Published:** 2012-01-12

**Authors:** Maysa F. Zampa, Inês Maria de S. Araújo, José Ribeiro dos Santos Júnior, Valtencir Zucolotto, José Roberto de S. A. Leite, Carla Eiras

**Affiliations:** ^1^Instituto Federal de Educação, Ciência e Tecnologia do Piauí (IFPI), Campus Parnaíba, 64210260 Parnaíba, PI, Brazil; ^2^Biotec, Núcleo de Pesquisa em Biodiversidade e Biotecnologia, Universidade Federal do Piauí (UFPI), Campus Ministro Reis Velloso (CMRV), 64202020 Parnaíba, PI, Brazil; ^3^Departamento de Química, Centro de Ciências da Natureza (CCN), Universidade Federal do Piauí (UFPI), 64049550 Teresina, PI, Brazil; ^4^Grupo de Biofísica Molecular Sérgio Mascarenhas, Instituto de Física de São Carlos (IFSC), USP, 13560970 São Carlos, SP, Brazil

## Abstract

The antimicrobial peptide dermaseptin 01 (DS 01), from the skin secretion of *Phyllomedusa hypochondrialis* frogs, was immobilized in nanostructured layered films in conjunction with nickel tetrasulfonated phthalocyanines (NiTsPc), widely used in electronic devices, using layer-by-layer technique. The films were used as a biosensor to detect the presence of dopamine (DA), a neurotransmitter associated with diseases such as Alzheimer's and Parkinson's, with detection limits in the order of 10^−6^ mol L^−1^. The use of DS 01 in LbL film generated selectivity in the detection of DA despite the presence of ascorbic acid found in biological fluids. This work is the first to report that the antimicrobial peptide and NiTsPc LbL film exhibits electroanalytical activity to DA oxidation. The selectivity in the detection of DA is a fundamental aspect for the development of electrochemical sensors with potential applications in the biomedical and pharmaceutical industries.

## 1. Introduction

Nanomaterials are causing a great impact on electrochemical biosensors development. Nanotechnology brings new possibilities for biosensors construction and for developing novel electrochemical bioassays [1].

Due to the increased use of organic molecules, the LbL technique has been widely employed in manufacturing ultrathin films with potential application as biosensors [2–6]. The ultrathin films' technique has many advantages, since it allows the construction of structures that present different chemical properties than those encountered in the originating materials [4–6].

A key feature for LbL films, in particular, is the incorporation of sulfonated groups to metallic phthalocyanines [7–9]. The electrodes modified with PAH/FeTsPc LbL films displayed electroactivity but were not suitable for dopamine detection. The development of voltammetric sensors for the detection of neurotransmitters, as dopamine (DA), in the extracellular fluid of the central nervous system has received much attention in the past few decades due to role in Parkinson disease [10, 11]. The electrochemical methods have advantages over others because they allow the detection of neurotransmitters in living organisms [12].

However, the coexistence of ascorbic acid (AA) with a concentration of 100–1000 times higher than that of DA greatly challenges the electrochemical strategy for DA detection. It was observed that AA could be easily oxidized at a potential close to the DA and the species formed could lead to oxidation of AA, as well as the reaction sites on the electrode surface could be easily blocked by the product of AA oxidation [13].

The electrochemical analysis of serum with a traditional solid electrode, such as glassy carbon, suffers from the problem of an overlapped oxidation potential between AA and DA. Moreover, there is no reversible electrochemical kinetics, so the fouling effect on the electrode surface by the oxidized AA could result in poor selectivity and reproducibility. Therefore, direct quantification of the AA concentration by the electrochemical method is difficult [14, 15].

Amphibian skin is an important source of gene-encoded AMPs, with more than half of ~900 eukaryotic peptides described to date isolated from South American Hylidae or European, Asian, or North American Ranidae [16]. The dermaseptins are a superfamily of AMPs that are produced in the skin of Hylidae and Ranidae frogs [17]. These peptides share a signature pattern consisting of a conserved Trp residue at position 3 and an AA(A/G)KAAL(G/N)A consensus motif in the midregion, which gives these molecules a cationic characteristic.

In this work we used a dermaseptin called DS 01, collected from the skin of *Phyllomedusa oreades* and *P. hypochondrialis* frogs. This molecule has demonstrated highly antibacterial activity against gram-negative and gram-positive bacteria as well as against many protozoans [17, 18]. The cationic features mainly refer to the ability of dermaseptins to exploit differences in lipid composition of the protozoan plasma membrane (PM). The PM of prokaryotes and lower eukaryotes is characterized by the presence of anionic phospholipids (PLs) at the outer leaflet, by the presence of certain sterols and to a lesser extent by a distinctive plasma membrane potential [19].

 Zampa et al. first demonstrated the immobilization of AMPs in electroactive thin films while maintaining bioactivity as shown in the enhanced detection of *Leishmania* by cyclic voltammetry [20].

In the present paper, we prepared stably assembled films using LbL method based on electrostatic interaction between the positively charged AMP layer, called dermaseptin 01 (DS 01), and negatively charged nickel tetrasulfonated phthalocyanine (NiTsPc) layer. We studied the electron transfer of DA and AA and further explored possible applications of this films for determination of DA in the presence of AA.

## 2. Experimental

### 2.1. Peptide Synthesis

The amidated DS 01 was synthesized on a Pioneer Synthesis System from Applied Biosystems (Framingham, MA, USA). Fmoc-amino acids and Fmoc PAL-PEG-PS resin were purchased from Applied Biosystems (Framingham, MA, USA). In order to purify the peptide, the use of a preparative C18 column (Vydac 218 TP 1022, Hesperia, CA, USA) on a HPLC system Class LC-10VP (Shimadzu Corp., Kyoto City, Japan) was required. Molecular mass (2793.6 Da) and sample purity were checked by MALDI-TOF MS. The final purification step of this synthetic peptide was performed by RP-HPLC on a Vydac 218 TP 54 (Hesperia, CA, USA) analytical column.

### 2.2. Solutions for LbL Depositions

**Figure 1:**
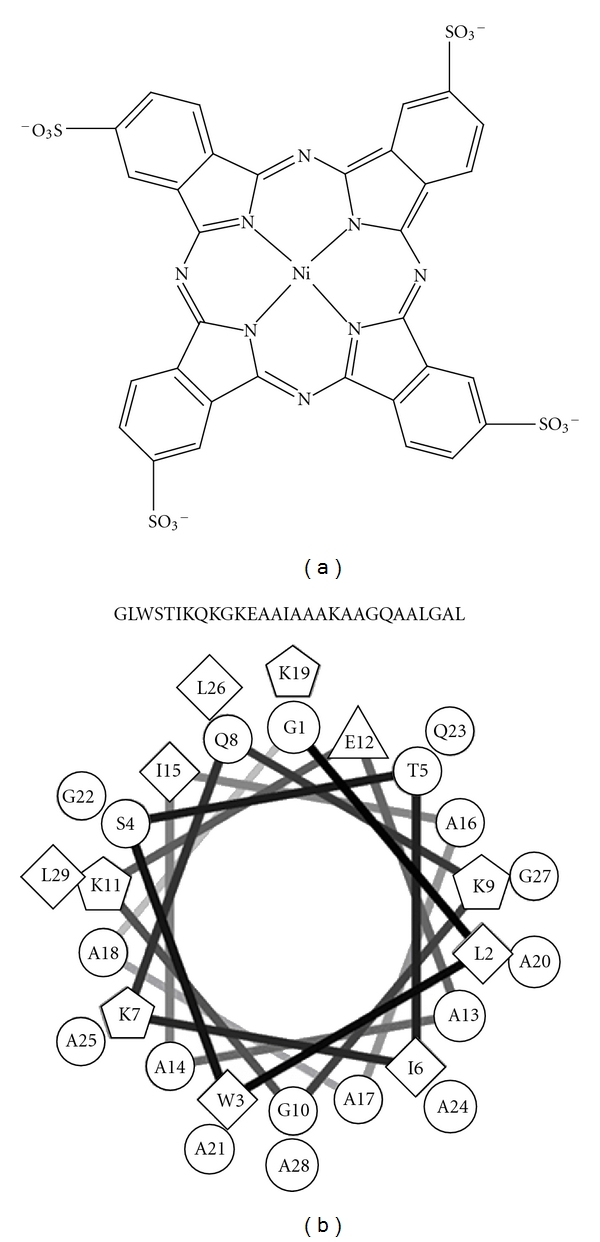
Chemical structures of materials used in LbL films: (a) nickel tetrasulfonated phthalocyanine (NiTsPc) and (b) amino acid sequence and helix-wheel plots of the antimicrobial peptide (DS 01) showing the amphiphilic character of the molecule.

The DS 01 solution used to construct the LbL films was prepared at a concentration of 3.58 × 10^−6^ mol L^−1^ in Milli-Q water, and the resulting solution had pH set at 5.6. Already NiTsPc, purchased from Aldrich Co. and used without further purification, was used at a concentration of 5.11 × 10^−4^ mol L^−1^ in HCl (pH 2.5) solution. The Figures 1(a) and 1(b) show the structures of NiTsPc and DS 01, respectively. DA and AA stock solutions were prepared at 10^−3^ mol L^−1^ concentrations, both commercially purchased from Aldrich Co..

### 2.3. Multilayer Deposition

**Figure 2:**
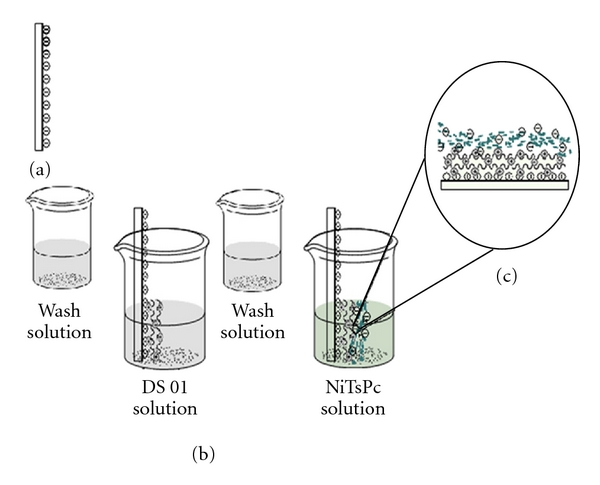
LbL film assembly process: scheme of film formation according to the LbL technique: (a) bare substrate, (b) deposition of the first layer of DS 01 AMP, (c) first bilayer formed (ITO/(DS 01/NiTsPc)_1_).

DS 01-containing films were produced combining the AMP with NiTsPc. At pH 5.6, DS 01 bears a positive net charge, in a way that interactions between the anionic NiTsPc and cationic DS 01 were primarily electrostatic. Immobilization was carried out in a LbL fashion upon the alternate immersion of ITO (indium tin oxide- (ITO)-covered glass plates) in solutions containing DS 01 and NiTsPc, respectively, for 5 min followed by immersion in the washing solution (HCl pH 2.5). An illustration showing film fabrication and immobilization is shown in Figure 2. After each immersion step, the film was dried using a nitrogen gas flow. The architecture investigated was ITO/(DS 01/NiTsPc)_3_ where 3 is the number of bilayers. For comparison effect, ITO modified with one monolayer of NiTsPc was studied.

### 2.4. Cyclic Voltammetry

Cyclic voltammograms were collected with LbL films deposited onto ITO using a potentiostat Autolab PGSTAT 30 Eco Chemie and a three-electrode electrochemical cell. A 1.0 cm^2^ platinum foil was used as auxiliary electrode, and the reference electrode was an Hg/HgCl/KCl (sat.) (SCE). All the potentials were referred to SCE. The LbL films deposited on ITO plates were employed as working electrodes. Experiments were carried out using a solution of H_2_SO_4_ 0.05 mol L^−1^ at room temperature. Cyclic voltammetry was also employed for DA detection, with DA being added in the electrolytic solution in a concentration range from 0 to 1.96 × 10^−5^ mol L^−1^.

## 3. Results and Discussion

**Figure 3:**
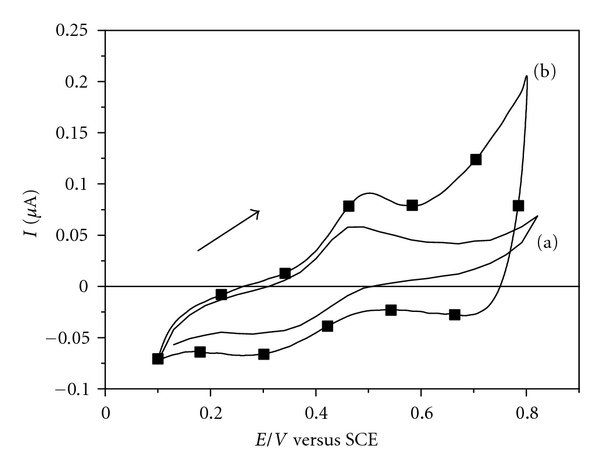
Electrochemical profiles of the films: cyclic voltammograms of (a) NiTsPc monolayer onto ITO substrate and (b) ITO/(DS 01/NiTsPc)_3_ film. Electrolyte solution H_2_SO_4_ 0.05 mol L^−1^, at 25 mVs^−1^.

To test the possibility of synergism between the molecules of DS 01 and NiTsPc, we compared the electrochemical profile of a NiTsPc monolayer (ITO/(NiTsPc)_1_) and the film containing three bilayers (ITO/(DS 01/NiTsPc)_3_), as shown in Figure 3. The electrical response signal of NiTsPc monolayer was detected. It is assumed that this low response in the electrical signal is a result of a small amount of NiTsPc electroactive molecules adsorbed in the unique monolayer on ITO surface.

The anodic process observed around +0.5 V may be related to the oxidation of nickel metal center [Ni(II)/Ni(III) + *e* ^−^] [21, 22], and the corresponding reduction process can be detected at +0.3 V. Figure 3 also illustrates the cyclic voltammogram of the ITO/(DS 01/NiTsPc)_3_ film, which shows two reduction processes at +0.3 V and +0.7 V, while the latter may be related to the reduction of the phthalocyanine macrocycle. The film shows a new electrochemical profile, different from that observed for the NiTsPc monolayer, suggesting that materials interact at the molecular level and displays new properties.

Voltammetric experiments demonstrated the stability of ITO/(DS 01/NiTsPc)_3_ film, which after successive scanning cycles continued to exhibit redox processes at the same level of electrical current (data not shown).

**Figure 4:**
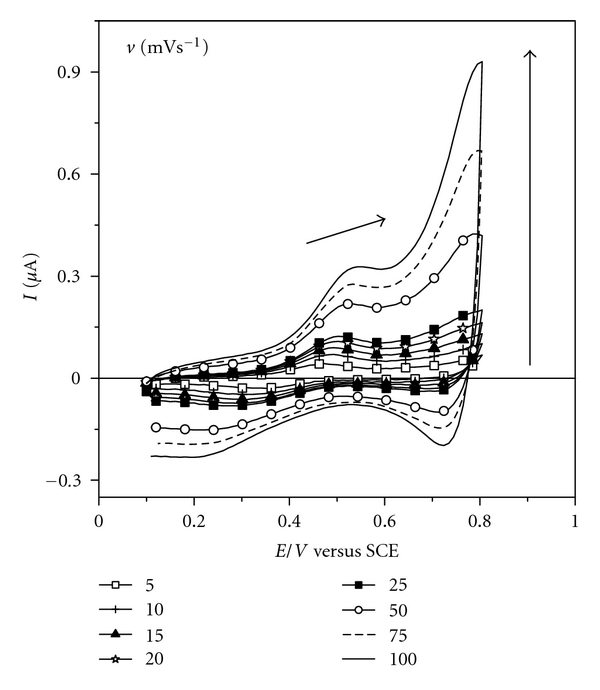
Study of the redox process of the film: cyclic voltammograms for ITO/(DS 01/NiTsPc)_3_ film at different scanning speeds. Electrolyte solution H_2_SO_4_ 0.05 mol L^−1^.

 The study of electrochemical activity of the film at different scan speeds is important to identify the mechanism that governs the redox process of the material. To perform this experiment, scanning cycles were recorded in scan speeds ranging between 10 and 100 mV s^−1^, as observed in cyclic voltammograms in Figure 4.

The linear relationship between anodic peak current and scan rate, which could be expressed by *I*_*p*_(*μ*A) = (0.0030 ± 0.0002) × 10^3^ *v* (V s^−1^) + (0.044 ± 0.008) (*r* = 0.992, *n* = 8), indicates that the reaction is governed by a mechanism of charge transfer between neighboring redox centers and the surface of ITO substrate, called electron hopping. This electron transfer process is associated with the existence of molecules immobilized on the substrate surface [23, 24].

The identification of important features of ITO/(DS 01/NiTsPc)_3_ film, such as electrochemical profile, stability, and the mechanism that governs the electronic charges, led to the application experimenting of DA detection and its interferent AA, testing the use of the film as an electrochemical biosensor.

**Figure 5:**
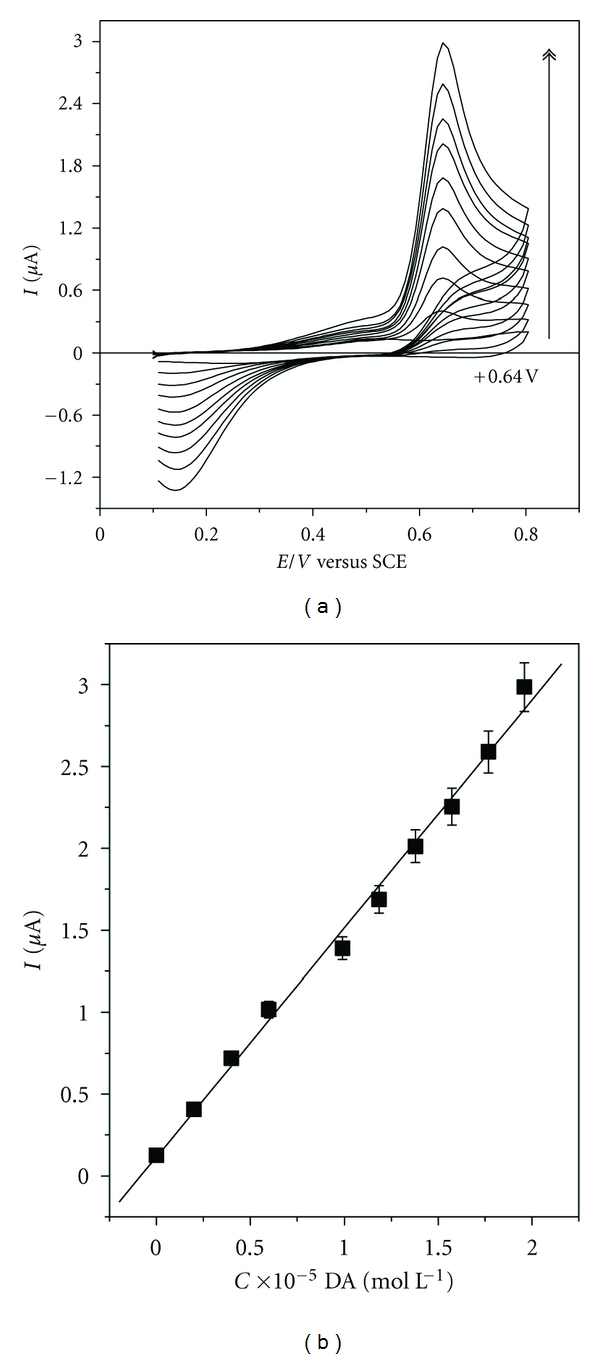
Dopamine detection test: (a) cyclic voltammograms for DA detection at concentrations ranging from 0 to 1.96 × 10^−5 ^mol L^−1^, in H_2_SO_4_ 0.05 mol L^−1^ at 25 mVs^−1^, for ITO/(DS 01/NiTsPc)_3_ film. (b) Calibration curve (*I* versus *C*) for DA detection.

Figure 5(a) reports the voltammograms obtained for ITO/(DS 01/NiTsPc)_3_ film after successive additions of aliquots of DA stock solution. The concentration of DA in the electrochemical cell ranged from 0 to 1.96 × 10^−5 ^mol L^−1^. It was observed that DA oxidation occurred at +0.64 V, and the electrical signal response increased linearly with the increase of analyte concentration in the electrolyte solution (Figure 5(b)). The linear regression equation was *I*_*p*_(*μ*A) = (1.40 ± 0.04) × 10^5^ *C* (mol L^−1^) + (0.12 ± 0.05) (*r* = 0.997, *n* = 10).

Using the information above, we calculated the detection limit (DL) which was 1.665 × 10^−6 ^mol L^−1^ [25]. The DL indicates the lowest concentration of analyte that can be detected by the biosensor without interference from noise caused by current equipment. The results of this work indicate that the biosensor is able to detect DA concentrations in a limit employed by the industry, for example, the pharmaceutical field.

**Figure 6:**
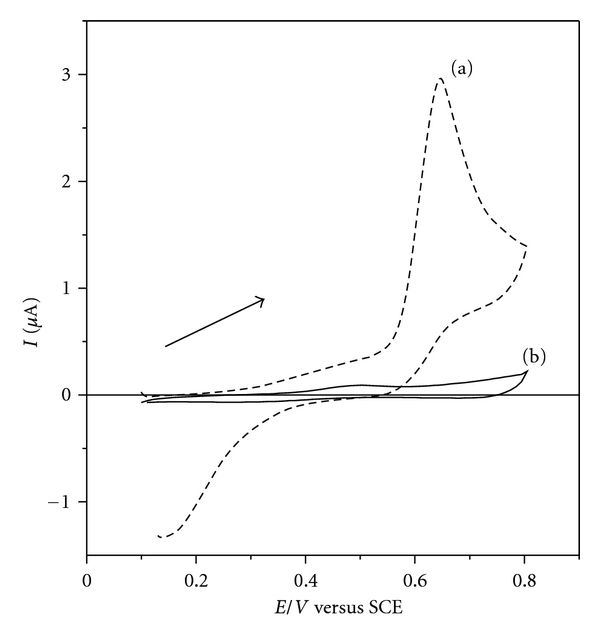
Study of the interaction between dopamine and film active sites: comparison between the electrochemical profile of ITO/(DS 01/NiTsPc)_3_ film in (a) presence of DA (1.9608 × 10^−5^ mol L^−1^) and in (b) DA absence, electrolyte solution H_2_SO_4_ 0.05 mol L^−1^, at 25 mVs^−1^.

Another important study was to assess whether DA permanently binds to active sites of the film after the detection test.  Figure 6 shows the cyclic voltammogram of the film at the maximum concentration of DA (1.9608 × 10^−5 ^mol L^−1^) in electrolyte solution. Then, the film was applied to potential scans in the electrolyte solution free of DA. The electrochemical profile of ITO/(DS 01/NiTsPc)_3_ film in this condition was shown in Figure 6 and indicates that the DA oxidation process at +0.64 V is no longer observed. Thus, the results suggest that the DA does not bind to the biosensor active sites permanently, because, after washing the biosensor in the electrolyte solution, there is no evidence of DA presence there.

**Figure 7:**
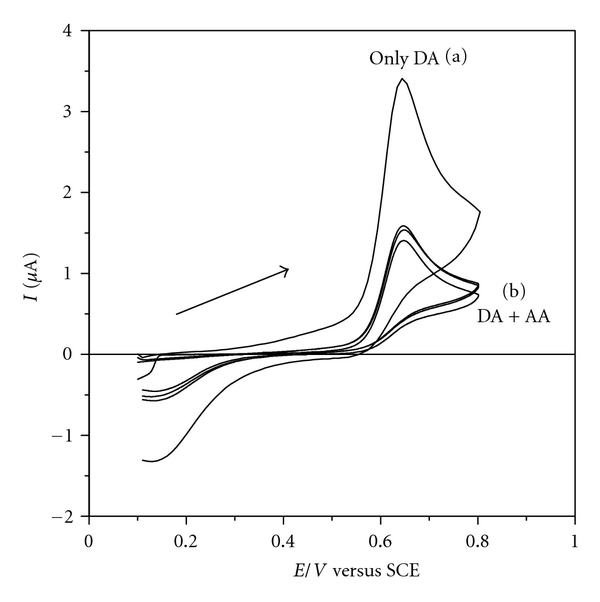
Interferent detection test: cyclic voltammograms for ITO/(DS 01/NiTsPc)_3_ film in (a) 1.9608 × 10^−5 ^mol L^−1^ of DA and (b) in presence of DA (1.9608 × 10^-5 ^mol L^−1^) and AA simultaneously, in different proportions *C*_DA_/*C*_AA_: 1 : 1, 1 : 2, and 1 : 3. Electrolyte solution H_2_SO_4_ 0.05 mol L^−1^, at 25 mVs^−1^.

As discussed above, it is necessary to study the electrochemical profile of the film in the presence of the analyte, DA, and its interferent AA, at the same time.  Figure 7 illustrates the cyclic voltammograms obtained for ITO/(DS 01/NiTsPc)_3_ film in the presence of DA and AA simultaneously in the following proportions *C*_DA_/*C*_AA_: 1 : 1, 1 : 2, and 1 : 3. It is observed that the voltammograms are identical, independent of AA concentration tested, exhibiting only a single oxidation process at +0.64 V and the same response level of electric current.

**Figure 8:**
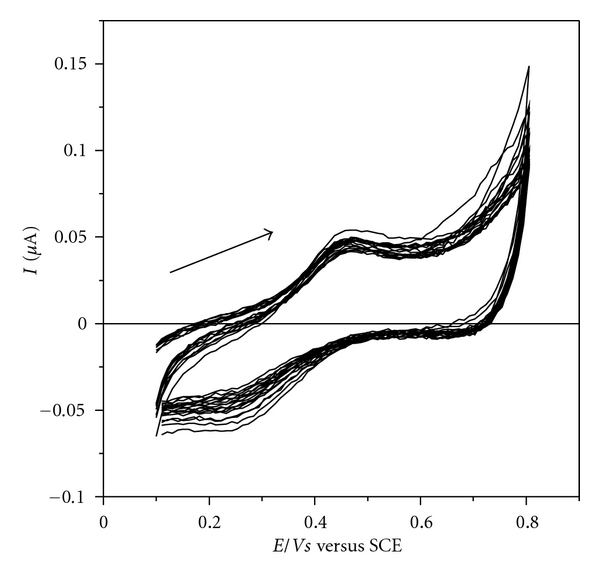
Ascorbic acid detection test: cyclic voltammograms for ITO/(DS 01/NiTsPc)_3_ film after successive additions of stock solution containing AA aliquots, in concentrations ranging from 1.235 × 10^−5^ to 20.000 × 10^-5 ^mol L^−1^. Electrolyte solution H_2_SO_4_ 0.05 mol L^−1^, at 25 mVs^−1^.

In this last stage of tests, the capability/ability of the biosensor in detecting only the presence of the interferent AA was studied. In this case, the experiments were conducted in an electrolyte solution without DA.  Figure 8 shows the cyclic voltammograms of the film after AA successive additions to the electrolytic solution, and there is no variation in electrical response signal; that is, the proposed biosensor does not detect AA alone. According to the results, it appears that the ITO/(DS 01/NiTsPc)_3_ film detects DA presence in the electrolytic medium which also contains the two analytes (AA and DA); however, the film is unable to detect the concentrations of DA and AA. It is assumed that AA probably adsorbs on electroactive film sites, blocking the electrical signal response of DA.

According to these discussions, the biosensor can be considered selective for DA, since the selectivity is the ability of a method to determine the analyte reliably in the presence of other substances that may interfere in the determination. Selectivity is an important parameter for the electroanalytical method validation.

Previous results observed for LbL films of POMA or gum containing NiTsPc [26, 27] indicated the overlapping oxidation processes of DA and AA, yielding in the tested films the characteristic of not being selective.

Zampa et al. [28] built biosensors for DA detection using the natural chicha gum (*Sterculia striata*) in tetralayers fashion with LD at approximately 10^-5 ^mol L^−1^. The process of DA detection in this biosensor was considered irreversible and governed by a diffusional process. No tests were performed to detect DA in the presence of AA.

In the present study, the biosensor ITO/(DS 01/NiTsPc)_3_ showed an even lower LD, about 10^-6 ^mol L^−1^, emphasizing that the presence of the DS 01 AMP along with NiTsPc was a decisive factor to confer the observed selectivity in DA and AA detection. The behavior of the film studied as a biosensor encourages the advance of application research of this nanostructured system in the biomedical and pharmaceutical areas.

## 4. Conclusions

Employing the LbL technique, a film containing the DS 01 AMP and NiTsPc macromolecules was constructed. DS 01 AMP is a biological material studied for prospective drugs while NiTsPc is a conductive material broadly utilized in researches in electronic devices. The ITO/(DS 01/NiTsPc)_3_ film was characterized as a stable system, able to detect DA in array bounds of 10^-6 ^mol L^−1^ besides being an selective electrode for AA. A selective electrode had not yet been developed by this research group. According to these results, it is expected that these films become objects of study in the important field of electrochemical biosensors.
